# Nonexposed endoscopic wall inversion surgery with sentinel node mapping for a high risk gastric lesion

**DOI:** 10.1055/a-2342-0221

**Published:** 2024-06-18

**Authors:** Alanna Ebigbo, Dmytro Vlasenko, Markus W. Scheppach, Sandra Nagl, Matthias Anthuber, Helmut Messmann

**Affiliations:** 1 Department of Gastroenterology, University Hospital Augsburg, Augsburg, Germany; 2 Department of Abdominal and Visceral Surgery, University Hospital Augsburg, Augsburg, Germany

Endoscopic submucosal dissection (ESD) is the standard approach for the management of early gastric cancer (EGC) 1 . However, in some situations, such as in borderline lesions, a combined laparoscopic-endoscopic technique may be a valid option 2 . We report the case of an 85-year-old man with multiple comorbidities who presented to us for further management of an EGC in the gastric body.

Endoscopic and endosonographic assessment revealed an ulcerated lesion (Paris IIc) with a suspicion of at least deep submucosal invasion (T1b/T2 N0). Owing to his age and comorbidities, the patient refused a gastrectomy but agreed to an individualized approach. After multidisciplinary team discussion, we opted for nonexposed endoscopic wall inversion surgery (NEWS) with sentinel node mapping ( Video 1 ).

Nonexposed endoscopic wall inversion surgery in an elderly patient with a suspected T2 gastric cancer.Video 1

NEWS is a subcategory of laparoscopic-endoscopic combined surgery 2 whereby the laparoscopic surgeon performs an initial seromuscular incision around the lesion, inverts the entire lesion along with a spacer intraluminally, and then places a seromuscular suture. Finally, the endoscopist performs a full-thickness resection of the lesion, while taking care to spare the seromuscular suture placed by the laparoscopist 3 . This nonexposed approach prevents possible peritoneal spillage and at the same time allows for full-thickness resection.

Compared with laparoscopic wedge resection, NEWS enables minimal tissue resection, especially in difficult positions 4 . Early data on NEWS and sentinel node mapping have demonstrated their safety and efficacy 4 5 . Histopathology in our patient showed a moderately differentiated cancer (G2) with deep submucosal invasion of 600 µm. Three lymph nodes resected by sentinel node mapping were negative.

Endoscopy_UCTN_Code_CPL_1AJ_2AD_3AF

## Notes

Endoscopy E-Videos https://eref.thieme.de/e-videos
*E-Videos* is an open access online section of the journal *Endoscopy* , reporting on interesting cases and new techniques in gastroenterological endoscopy. All papers include a high-quality video and are published with a Creative Commons CC-BY license. Endoscopy E-Videos qualify for HINARI discounts and waivers and eligibility is automatically checked during the submission process. We grant 100% waivers to articles whose corresponding authors are based in Group A countries and 50% waivers to those who are based in Group B countries as classified by Research4Life (see: https://www.research4life.org/access/eligibility/ ). This section has its own submission website at https://mc.manuscriptcentral.com/e-videos .
